# Giant prolactinoma: case report and review of literature

**DOI:** 10.1186/2251-6581-12-3

**Published:** 2013-01-08

**Authors:** Masoud Rahmanian, Hamidreza Aghaei Meybodi, Bagher Larijani, Mohammad-reza Mohajeri-Tehrani

**Affiliations:** 1 EMRC (Endocrinology and Metabolism Research Institute), Shariati Hospital,Tehran university of medical science, North Karegar St, Tehran, Iran

**Keywords:** Giant prolactinoma, Cabergoline, Hypothyroidism

## Abstract

“Invasive giant prolactinoma” is a large prolactinoma (>4 cm in dimension) presenting with serum prolactin levels of >1000 ng/dL and mass related clinical symptoms. Here we report a patient with a giant prolactinoma presented with central hypogonadism, suppressed adrenal and thyroid function, supra sellar extension, visual field impairment and high prolactin level.

The patient was treated with cabergoline, levothyroxin and prednisolone. After 18 months, tumor size markedly reduced, associated with adrenal function and visual field improvement, but central hypogonadism and secondary hypothyroidism persisted.

Previous studies showed normalization of thyrotropin secretion after treatment but it remained low in our patient even after 18 months follow up.

## Background

Prolactinoma is the most common pituitary adenoma [1] which causes infertitity, menstrual irregularity and galactorrhea [2,3] in women and hypogonadism, decreased libido, infertility, erectile dysfunction and gynecomastia [4,5] in men.

In the present report we describe an unusuall giant prolactin – producing macroadenoma of pituitary and its response to cabergoline (a dopamine agonist).

## Case presentation

**Figure 1:**
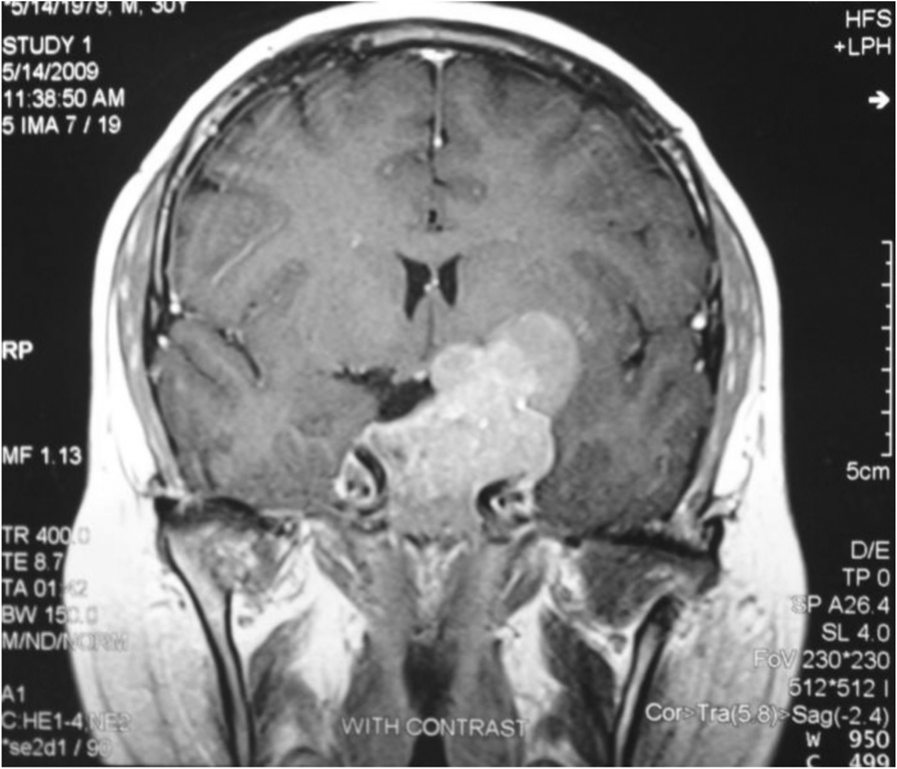
Huge macroprolactinoma with suprasellar invasion before treatment.

**Figure 2:**
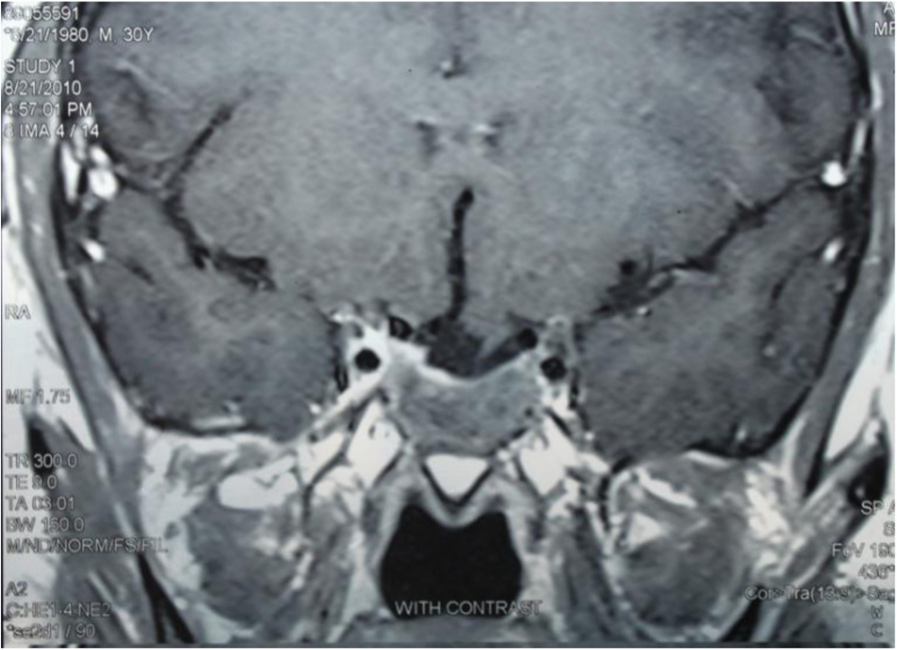
14 months after treatment; significant tumor shrinkage.

A 29 year – old married man was referred to us with headache, blurred vision, diplopia, decreased libido and weight gain from 12 months ago. He was married but did not have child. Laboratory evaluations showed prolactin levels as high as 12000 ng/mL (1.8–20) and further evaluations revealed central hypogonadism, secondary hypothyroidism, adrenal insufficiency and normal IGF1 level (Table 1). Pituitary MRI showed a large pituitary solid – cystic mass with suprasellar and left cranial fossa extension measuring 5.5 × 5.0 × 2.5 cm in dimension (Figure 1) and perimetry detected right side temporal hemianopia. Treatment with cabergoline (a dopamine agonist) 1 mg twice a week, levothyroxin 0.1 mg daily and prednisolone 7.5 mg resulted in visual improvement in one month. Prolactin level decreased to 222 ng/ml after 3 months but impotence and decreased libido didn’t show any improvement. Testosterone level was 1.32 ng/mL (2.8–8). He was started on testosterone (250 mg IM each 3 weeks) and cabergoline dose increased to 3 mg per week. One year after treatment, prolactin level reached to 165 ng/mL; ACTH stimulation test (Synacthen 0.25 mg IV injection and plasma cortisol measurement before, 30 and 60 min after administration) results were normal. So we discontinue prednisolone. Eighteen months later perimetry test showed normal results, prolactin level diminished to 71.5 ng/mL and tumor size markedly reduced on MRI to 2.2 × 2.3 × 1.2 cm in dimention (Figure 2) but central hypogonadism and secondary hypothyroidism persisted (Table 1).

**Table 1:** Hormonal evaluation before and after treatment

	**Normal range**	**Before treatment**	**After treatment**
**Prolactin**	1.8 – 20 ng/mL	12000	71.5
**IGF- I**	219 – 614.4 ng/mL	120	230
FSH	1.4 – 15.4 mIU/mL	0.6	3.1
**LH**	1.2 – 7.8 mIU/mL	0.8	2.7
**Testosterone**	2.8 – 8 ng/mL	<0.02	1
**Cortisole**	6.2 – 14.4 micg/dL	0.4	10
**Cortisole 30 min after ACTH injection**		4.4 μg/dL	22.2 μg/Dl
**Cortisole 60 min after ACTH injection**		5.2 μg/dL	34.1 μg/Dl
**ACTH**	7.2 – 63 pg/dL	8.8	
**T4**	5.1 – 14 micg/dL	5.5	8.2
**TSH**	0.5 – 5 mU/L	2.05	0.2

## Conclusion

Pituitary tumors larger than 4 cm in size are termed “giant adenomas” [6]. “Invasive giant prolactinoma” is defined as : 1) tumor diameter of >4 cm; 2) serum prolactin >1000 ng/mL; and 3) clinical symptoms induced by hyperprolactinemia or mass effect [7]. Most women lactotroph adenomas are microadenoma, however the men adenomas are usually larger [8]. Giant prolactinoma is rare and usually presents in men [9,10]. Complete surgical removal of giant tumor is difficult and biochemical cure is rare [11,12].

In prolactin – secreting macroadenemas, goals of treatment are to decrease tumor size, improve visual field defects and restore sexual function. Dopomine agonists are able to reach these goals with reducing tumor size [13,14]. In comparison with bromocriptine, cabergoline has fewer side effects and more positive effects at normalizing prolactin levels [15]. If visual field defect persists and chiasmal compression on MRI examination continues despite optimal medical treatment, surgery will be inevitable. But little is known about the role of dopamine agonist therapy in treatment of giant invasive prolactinomas. There were studies evaluating cabergoline (a dopamine agonist) in management of giant prolactinoma [14,16,17]. All of these studies showed that cabergoline is safe and well-tolerated and also suggested that cabergoline should be the first line of treatment for giant aggressive macroprolactionomas. In one study, cabergoline normalized prolactin levels in ten out of twelve patient and decreased significantly in the other two significantly. Visual field defect was present in nine patients at diagnosis which returned to normal in three of patients and improved in five after treatment. Tumor diameter responded to treatment with decrease of 47+/- 21% in size. Testosterone levels returned to normal in eight out of twelve patients. In this study only two of patients had secondary hypoadrenalism and hypothyroidism who improved after treatment [14]. Hypothyroidism and hypoadrenalism improve usually following normalization of prolactin level and tumor size in macroprolactinomas [14,15,18] but little is known about their recovery in giant prolactinoma. Hypogonadism persists in half of male patients with macroprolactinoma despite tumor shrinkage and normalization of prolactin levels [14,16,19,20]. Therefore testosterone replacement is required to maintain bone strength. Androgen can cause secondary rises in prolactin levels that postulated to central aromatization to estrogens [21].

In our case, tumor size markedly reduced and visual field and ACTH secretion recovered completely after 18 months of cabergline treatment but central hypogonadism and secondary hypothyroidism persisted. Somatotroph and gonadotroph cells are the most sensitive pituitary cells to injury secondary to sellar compression, but in this case thyrotroph cells also showed impaired function which did not recover after significant tumor shrinkage. With our best knowledge, there is not any report about this unusual response of giant prolactin-producing macroadenoma to cabergoline treatment in literature.

## Consent

Written informed consent was obtained from the patient for publication of this case report.

## Competing interests

The authors declare that they have no competing interest.

## Authors’ contribution

MR wrote the final version of manuscript, HAM read the case and comfirmed manuscript, BL supervise management of the case and approved manuscript, MRMT manage the patient and finalize the manuscript. All authors read and approved the final manuscript.
